# The impact of occupational fatigue on safety and health performance in the porcelain manufacturing industry: An analysis using the ELMERI index

**DOI:** 10.1016/j.dialog.2026.100283

**Published:** 2026-02-04

**Authors:** Mostafa Jafarizaveh, Razie Jafarizadeh, Maryam Esmaili, Akram Tabrizi

**Affiliations:** a Department of Occupational Health and Safety Engineering, Faculty of Health, Social Determinants of Health Research Center, Gonabad University of Medical Sciences, Gonabad, Iran; b Department of Epidemiology and Biostatistics, Faculty of Health, Gonabad University of Medical Sciences, Gonabad, Iran; c Student Research Committee, Department of Occupational Health and Safety Engineering, School of Public Health and Safety, Shahid Beheshti University of Medical Sciences, Tehran, Iran

**Keywords:** ELMERI index, Job fatigue, Safety, Public health

## Abstract

As a critical social determinant of health, occupational fatigue can be a key indicator for tracking mental health inequalities in working populations. A systematic assessment of fatigue and its impact on safety performance indicators is essential for developing effective preventive strategies in industrial settings. This study aimed to investigate the correlation between occupational fatigue and safety and health performance within an industrial sector in Gonabad, Iran. A descriptive-analytical, cross-sectional study was conducted among industrial workers. Data were collected using a demographic questionnaire, the Swedish Occupational Fatigue Inventory (SOFI) to quantify fatigue, and the ELMERI index to assess safety and health performance levels. Kolmogorov-Smirnov, *t*-test, Mann-Whitney, and Spearman's rank correlation coefficient tests were used for statistical analysis between variables. The study included participants with a mean work experience of 8.49 (±4.63) years. The mean SOFI score for occupational fatigue was 52.75, indicating a significant burden of fatigue. The mean ELMERI index score was 59.50, reflecting a moderate level of safety and health performance. A significant inverse correlation was found between occupational fatigue and safety performance (*r* = −0.40, *p* < 0.05), suggesting that higher levels of fatigue were associated with poorer safety and health outcomes. The findings demonstrate a significant negative relationship between occupational fatigue and safety performance, highlighting the detrimental impact of fatigue on workplace safety. These results underscore the need for implementing fatigue risk management systems and ergonomic interventions in the workplace. Future longitudinal research is recommended to elucidate further the causal mechanisms underlying this relationship.

## 1 Introduction

As a critical social determinant of health, fatigue can be a key indicator for tracking mental health inequalities in working populations. Fatigue is one of the common problems in developed countries, which has a high prevalence both in societies and in work environments. In general, fatigue is a multidimensional and complex issue that has different structures and components, and for this reason, achieving a single definition and Comprehensive is difficult [1]. Fatigue is actually a mental term that is described with physical fatigue, and rapid fatigue is a common complaint of people after a hard and long day at work [2]. There are different methods for classifying fatigue. Fatigue can be categorized into two types based on its duration: acute fatigue and chronic fatigue. Acute fatigue can be quickly alleviated through rest or lifestyle changes, whereas chronic fatigue is a condition that persists for more than a few months and does not improve with rest. Fatigue can also be divided into two types: mental fatigue, which relates to cognitive or perceptual aspects, and physical fatigue, which is associated with the performance of the motor system [3]. Physical fatigue is associated with a decline in muscle function and is characterized by a reduction in the strength or force needed for movement and muscle contraction. In contrast, mental fatigue involves a decrease in cognitive abilities such as concentration, reasoning, learning, and quick response [2]. A study conducted in the United States revealed that the economic impact of this phenomenon exceeds $100 billion annually [4].

In recent years, due to the relatively high prevalence of fatigue and its effects, including the impact on family life, the cardiovascular system, the creation of skeletal-muscular disorders, as well as efficiency and mental, physical and psychological performance [5], [6], [7], the occurrence of human error [8], [9]), the occurrence of accidents among workers [4], damage to memory and the power of decision-making and reasoning [10], increasing the risk of depression, anxiety and endangering the mental health of workers [8], absenteeism and disability [11]), this phenomenon has attracted the attention of most people. Fatigue and rapid burnout are critical concerns in applied occupational medicine that need to be addressed to better understand their impact on employees' health, performance, and safety [12]. Fatigue can cause errors and reduce performance by affecting people's performance, which can ultimately increase the risk of adverse consequences in safety [13]. Fatigue in nurses can cause an increase in errors such as medication errors, patient identification errors, as well as decreased performance and needle stick accidents [14].

Fatigue is a common phenomenon and the result of physical and mental activities and emotional stress that depends on environmental and individual factors [15]. Workload-related factors and the nature of job tasks, such as monotony, boredom, and lack of stimulation, can contribute to both physical and mental fatigue, increasing safety risks [16]. Among the environmental causes of fatigue, factors such as high heat, inappropriate lighting, and high levels of noise and vibration can be mentioned [17], [18]. Fatigue, as a concept, connects various factors that can contribute to fatigue with several safety-related outcomes [19]. Today, work-related fatigue has gained significant attention due to its negative impact on both individuals and productivity, with many asserting that fatigue is becoming an increasing concern for health and safety.

The main goal of occupational health and safety is to minimize incidents and accidents related to workplace safety. Considering the various consequences of workers' fatigue in work environments, assessment of fatigue and safety level is one of the first and most basic measures.

One of the methods of evaluating the safety level in the workshop is the ELMERI index. The ELMERI index is a safety and reliability monitoring tool for manufacturing industries and for any company in any sector and any size, and this method performs quickly and calculation-free evaluation [20]. The safety index method, which is based on observations, has been used from 2002 to 2005 both in workplace inspections and for operational monitoring of the safety status of workplaces of companies with different types of economic activities [21]. The ELMERI index is a preventive method based on surveying of the work environment, working conditions and workers' behavior, and this method can provide information and the level of effectiveness of occupational health and safety management systems. The ELMERI index can be used as a feedback tool for the development of safe behaviors, based on all work areas in a work environment or a representative sample [22].

The studies conducted on the phenomenon of fatigue are mostly aimed at assessment of the relationship between fatigue and demographic characteristics, the rate of accidents and musculoskeletal disorders. Studies have shown that various factors can affect people's fatigue and fatigue can cause changes in people's performance and cause a decrease in safety level and an increase in errors and risks in the work environment, and considering that the ELMERI index can Measure and assess the level of safety in various dimensions of safe behaviors, industrial hygiene, Housekeeping, machine safety, ergonomics, passageways, fire safety and first aid, in order to reduce accidents in the workplace, understanding of the relationship between the dimensions Various safety and health dimensions with people's work fatigue can be very productive and useful in this way. Therefore, in the present study, the relationship between job fatigue and different dimensions of the ELMERI index was investigated.

## 2 Research methods

The present study is a cross-sectional study. The participants in this study are employed in a Chinese manufacturing industry. The number of employees in this industry is 500, and the sample size was calculated as 105 using the following formula with a 95% confidence interval (Eq. 1). The samples were selected by simple random sampling [23].

(1) $$n=\frac{z_{1-\frac{\propto }{2}}^{2}\sigma^{2}}{d^{2}}\;$$

The criteria for entering this study were to have at least six months of work experience, a fixed duty in a working period, complete physical and mental health, and not having a second job.

**Fig. 1:**
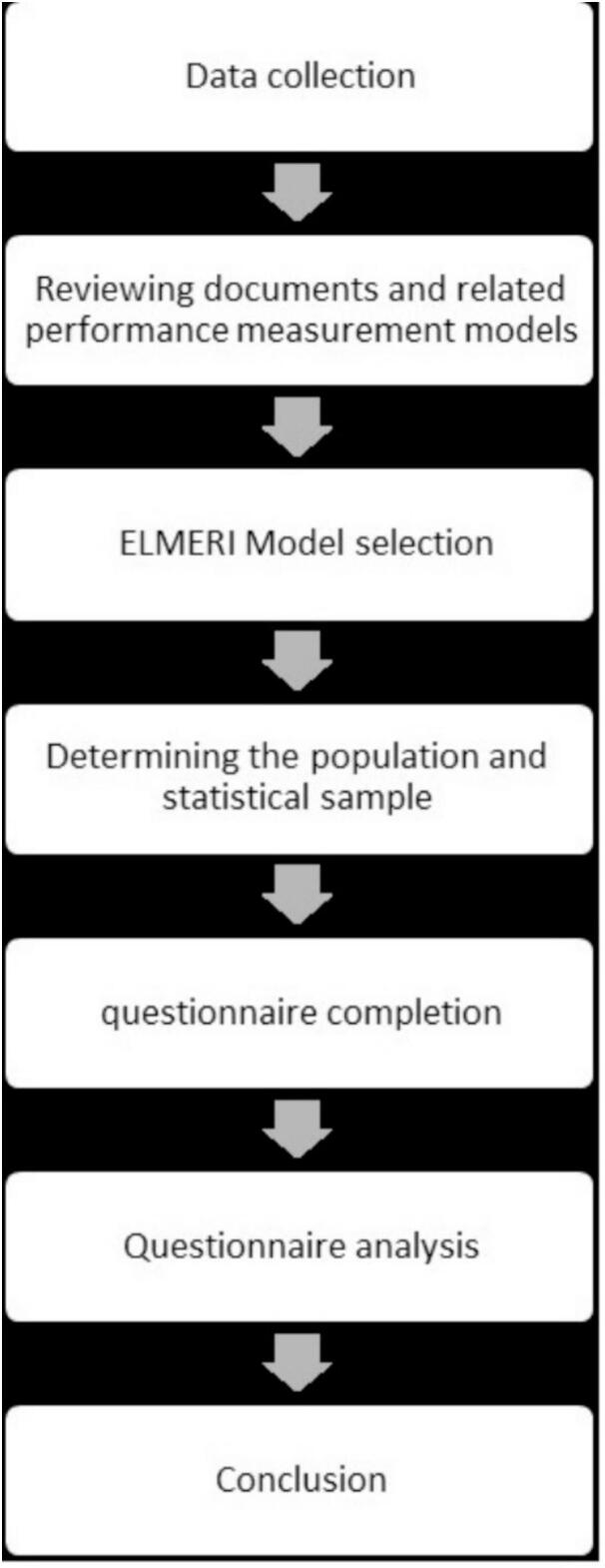
Implementation steps of ELMERI method.

In this study, after the Coordination with relevant industry management, taking into account ethical considerations, the questionnaires were completed. First, the demographic questionnaire and the Swedish Occupational Fatigue Inventory (SOFI) were completed. The SOFI questionnaire was designed and presented by Asber et al. in 1952. This questionnaire is a multi-dimensional tool that is used to measure the quality and intensity of acute fatigue and is able to evaluate psychological and physical aspects [6]. In many studies on different jobs, the SOFI questionnaire has been studied and it is known to be a reliable tool, and its reliability and validity have been found to be appropriate [56]. The SOFI-20 questionnaire includes five dimensions: “lack of energy,” “physical effort,” “physical discomfort,” “lack of motivation,” and “sleepiness,” with each dimension assessed through four items [24]. According to the initial versions of the SOFI questionnaire, in order to have more sensitivity in the measurement, each item was rated using an 11-point Likert scale from zero (not at all) to 10 (very much agreement) [25]. In total, this questionnaire has 20 questions that are answered by the individual. Ultimately, the answers obtained from the 20 questions will be added together, and the score range of this questionnaire varies between zero and 200 points. In this study, the ELMERI index was used to calculate the level of safety and health. The work steps are shown in Fig. 1.

Data for the ELMERI index were collected through direct observation by the research team, not through self-reports from the workplaces. To ensure reliability, two trained occupational health experts conducted the observations. Prior to data collection, both observers participated in a training session to establish a common understanding of the checklist items and scoring criteria, thereby minimizing inter-rater variability. The ELMERI method is based on observing all aspects of the safety and health of the work environment and the safety behaviors of the workers. Observations that are considered in the ELMERI index include safe behaviors, Housekeeping, machine safety, industrial hygiene, ergonomics, passageways, fire safety and first aid. In order to calculate the index, the mentioned items are observed in each work station and then, if the minimum safety and health standards of the work environment are observed, they are scored as correct observations and otherwise as incorrect observations. If any of the above seven groups are not observed for a work station, the expression of non-observation should be considered for it, and at the end, based on the number of correct observations and incorrect observations, according to the following formula, the ELMERI index score is determined. The values  of this index vary from zero to 100 and if the index score is 60 or more, it is considered as an acceptable level of safety and health. The equation for calculating the ELMERI index is as follows:

(2) $$\text{ELMERI}=\frac{\mathrm{correct}}{\mathrm{correct}+\mathrm{in correct}\;}\times 100$$

The data were analyzed using SPSS version 16.0, which was used for descriptive statistics, the Kolmogorov-Smirnov (K—S) test, the Independent Samples *t*-test, the Mann-Whitney *U* test, and Spearman's rank correlation coefficient.

## 3 Results

The results of checking the qualitative variables from the demographic questionnaire are shown in Table 1. According to the observed frequency, 75.2% of people are men and 24.8% are women. Most of the participants have a diploma level of education (59%) and also 93.3% of people are married and only 6.4% are single.

**Table 1:** Some demographic information of people participating in the study (*n* = 105).

Variable	The Basis of Variable Grouping	Number	Percent
Gender	Female	26	24.80
	Male	79	75.20
	Total	105	100
Education	Less than diploma	25	23.80
	Diploma	62	59
	up to BS	18	17.10
Marriage status	Single	7	6.70
	Married	98	93.30
	Total	105	100

Descriptive analysis of job fatigue questionnaire and ELMERI index results are shown in Table 2. According to Table 2, the study population is relatively homogeneous and stable in terms of age and work experience. The mean age of the participants was approximately 34 years, and their mean work experience was about 8.5 years. The mean Job Fatigue Score was found to be 52.75. The high standard deviation obtained indicates that job fatigue varies greatly among individuals; some participants reported very low fatigue scores, while others reported very high scores. Furthermore, the mean ELMERI Index Score was 59.5. Despite the homogeneity of the population in terms of age and work experience, the reported level of job fatigue among individuals is highly variable and dispersed. This is a significant finding, suggesting that factors beyond age and work experience (such as individual conditions, type of work unit, work pressure, managerial support, etc.) play a role in the development of job fatigue.

**Table 2:** Descriptive analysis results of quantitative variables studied.

Variable	SD ± Mean	Median	Range
Age	34.18 ± 5.59	34	25
Work experience	8.49 ± 4.63	8	21
Job fatigue score	52.75 ± 29.41	45	133
ELMERI Index score	59.50 ± 12.31	60	57

In Table 3, the relationship and correlation between job fatigue score and ELMERI index and its dimensions, as well as age and work history, which were investigated by Spearman's test, are given. As observed, there is a significant correlation between the job fatigue score and the ELMERI score. Additionally, a noteworthy relationship was found between the job fatigue score and the ELMERI dimensions, including safe behaviors, machine safety, industrial hygiene, and ergonomics. Also, job fatigue has the highest correlation with ELMERI index and only two dimensions of Housekeeping and fire safety and first aid did not show significant correlation with job fatigue. No significant relationship was observed between job fatigue score and work experience and age.

**Table 3:** The results of Spearman's correlation test to check the correlation between the fatigue score with different dimensions of the ELMERI index, age and work experience in the study subjects (*n* = 105).

Variable		*P*-value	Correlation Coefficient(r)
Total ELMERI index		*p* < 0.001	−0.400
ELMERI different dimensions	Safe behaviors	0.005	−0.0.270
	Housekeeping	0.89	−0.013
	Machine safety	0.013	−0.337
	Industrial hygiene	0.005	−0.255
	Ergonomics	0.009	−0.255
	Fire safety and first aid	0.139	−0.138
Work experience		0.366	0.089
Age		0.710	−0.037

In Table 4, the values of different dimensions of fatigue score with acceptable and unacceptable levels of ELMERI index were tested and compared using the Mann-Whitney test and *t*-test, And the results showed that there is a significant difference in the job fatigue score in terms of lack of energy, physical effort, physical discomfort and sleepiness in two levels, but no significant difference was observed in the dimension of lack of motivation.

**Table 4:** The average score of job fatigue questionnaire dimensions based on the ELMERI index level in the study subjects (*n* = 105).

Different Dimensions of Job Fatigue	Mean (SD)	Un acceptable level of ELMERI index mean (SD)	Acceptable level of ELMERI Index Mean (SD)	P-value
Lack of energy⁎	10.13(9.27)	13.76(9.46)	6.7(7.71)	0.0001
Physical effort⁎	6.93(6.52)	12.15(7.32)	7.83(4.85)	0.002
Physical discomfort⁎⁎	11.52(8.41)	14.86(8.69)	8.37(6.84)	0.0001
Lack of motivation⁎	12.03(7.11)	10.82(8.32)	13.18(5.58)	0.060
Sleepiness⁎	9.13(8.31)	11.76(9)	6.67(6.79)	0.003

⁎ Comparison made using the Mann-Whitney U test.

⁎⁎ Comparison made using the independent samples *t*-test.

In Table 5, the difference between job fatigue score with marriage and gender was tested using the Mann-Whitney test. No significant difference was observed between the job fatigue score in the variables of gender and marriage.

**Table 5:** The average job fatigue score based on gender and marriage in the study subjects (*n* = 105).

Variable			SD ± Mean	*P*-value
Job fatigue score	Gender	Female	56.23 ± 31.84	0.585
		Male	51.60 ± 8.69	
	Marriage status	Single	51.28 ± 25.93	0.959
		Married	52.85 ± 29.76	
	Education	Diploma	54.28 ± 27.18	0.871
		Less than a diploma	52.04 ± 29.13	
		up to BS	53.11 ± 34.67	

## 4 Discussion

The present study aimed to investigate the relationship between occupational fatigue and the ELMERI index. The results of this study showed that there is a significant relationship between occupational fatigue and the ELMERI index, which is one of the methods for measuring the level of safety and health, with a correlation of −0.4. In a study that was conducted to investigate the relationship between fatigue and sleepiness and the rate of accidents in a group of workers, a significant relationship between fatigue and sleepiness and the rate of accidents was observed [26] and the results of this study are consistent with the present study. Fatigue assessment is a more accurate factor for preventing work accidents compared to sleepiness. In the current study, a significant difference was observed between sleepiness, which is one of the dimensions of job fatigue, and the level of safety and health based on the ELMERI index. Also, in the study of Swaen [27] and the Ministry of Minerals and Energy of Australia [28], the impact of fatigue on accidents and incidents at work was well shown.

The findings of this study clearly indicate a significant relationship between safe behaviors and fatigue, with higher levels of fatigue leading to a decline in safe behaviors among individuals. In a study by Taherpour et al. [29], the results indicated that fatigue indirectly influences workers' safety risk perception through factors such as safety attitude and risk awareness. In this study, it was well shown that an increase in the level of fatigue leads to a more negative safety attitude, which can be expected because a negative safety attitude leads to an increase in unsafe behaviors. On the one hand, a negative safety attitude leads to a decrease in the perception of safety risk among workers and an increase in accidents among workers. This study and the current study clearly show the role of fatigue on unsafe behaviors. However, in the study of Hong J [30], safety incidents among nurses were investigated and the results showed that there is no difference in the frequency of safety incidents or medication errors between two-shift and three-shift nurses, and in order to investigate the impact of fatigue on human errors, a comprehensive and more detailed study is needed.

The results of this study show that there is a significant difference between different dimensions of fatigue such as physical discomfort, lack of energy, physical effort and sleep with the level of safety and health in the ELMERI index, which is consistent with the results of the study by Mirzaei et al. In which it was stated that there is a relationship between the number of minor errors and the five dimensions of fatigue, and as fatigue increases, the number of minor errors also increases.

Ann Williamson's study [31] also examined the relationship between fatigue and safety, focusing on three main factors thought to contribute to fatigue: circadian rhythm, sleep homeostasis, and work-related effects. The findings revealed that work-related factors significantly impact fatigue and performance. Factors contributing to fatigue negatively affect both performance and safety outcomes. There is strong evidence that fatigue undermines safety, and it is crucial to manage both fatigue and its causes carefully. This study also strongly supports the results of the current research. The results indicate a significant correlation between the industrial hygiene dimension, including factors such as noise, lighting, ventilation, air quality, and chemicals, and job fatigue. In the studies of Mirzaei [32] and Saremi [33], the results showed that the level of fatigue among people who are exposed to noise is higher than others, and there is a significant difference between the two.

Also, in a study [34], the results indicated that exposure to noise and certain air pollution components, particularly O3 and PM10, increases fatigue. This finding aligns with the current study, which highlights the role and impact of the work environment on job fatigue.

Based on the results, it was determined that the ergonomic factors of the workplace have a significant relationship with job fatigue, and increasing ergonomic levels in the workplace reduces fatigue in people, and this indicates the role of ergonomics in causing fatigue in the workplace. as well as in other studies [23] [35] [36] There is a significant relationship between ergonomic interventions and the prevalence of skeletal and muscular disorders and job fatigue.

The results of this study showed that there was no relationship and significant difference between the variables of age, work experience, marriage and education with job fatigue, which is consistent with the studies of Karimi [37] and QasemKhani [38]. In Bezazan's study [12], the results showed that there is a significant relationship between marriage and education with fatigue. Despite the significant differences between the variables, this study demonstrated that personal and occupational characteristics have a minimal impact on fatigue and mental disorders.

However, in other studies by Azad [17], Halvani [39], Chubineh [6] and Boltmand (41), the relationship between education level and fatigue has been found to be significant. In the study of Halvani [39] and Chubineh [6], age and marriage did not have a significant relationship with fatigue, which is consistent with the present study. The findings of this study highlight the clear need for additional research to address key unresolved questions regarding the connection between fatigue and safety and health performance.

This study is subject to certain limitations that should be considered when interpreting the findings. The primary limitation stems from its cross-sectional design, which, while revealing significant associations, precludes definitive causal inferences between the variables. Establishing causality requires future longitudinal research. Furthermore, while simple random sampling was employed to enhance representativeness, and the sample size was calculated with a 95% confidence level, the relatively modest sample size (*n* = 105) may still limit the generalizability of the results to the broader workforce in different contexts or with diverse characteristics. Future studies with larger sample sizes are recommended to confirm and strengthen the external validity of these results.

## 5 Conclusion

Consequently, we contend that fatigue must be reconceptualized from an individual worker's issue to a systemic organizational risk. Managers and safety officials are urged to adopt objective tools like the ELMERI index for continuous monitoring and to implement targeted strategies aimed at fatigue mitigation. Future studies should focus on validating these findings in larger cohorts and designing intervention-based models to further solidify the evidence base for these recommendations.

## CRediT authorship contribution statement

**Mostafa Jafarizaveh:** Writing – original draft, Visualization, Validation, Supervision, Software, Resources, Project administration, Methodology, Investigation, Funding acquisition, Conceptualization. **Razie Jafarizadeh:** Writing – review & editing, Formal analysis. **Maryam Esmaili:** Writing – review & editing, Data curation. **Akram Tabrizi:** Writing – review & editing, Writing – original draft, Visualization, Validation, Supervision, Software, Resources, Project administration, Methodology, Investigation, Conceptualization.

## Declaration of competing interest

The authors state that there is no conflict of interest in the present study.
